# Thoracic and cutaneous sarcoid-like reaction associated with anti-PD-1 therapy: longitudinal monitoring of PD-1 and PD-L1 expression after stopping treatment

**DOI:** 10.1186/s40425-018-0372-4

**Published:** 2018-06-13

**Authors:** Léa Paolini, Caroline Poli, Simon Blanchard, Thierry Urban, Anne Croué, Marie-Christine Rousselet, Sarah Le Roux, Nathalie Labarrière, Pascale Jeannin, José Hureaux

**Affiliations:** 1 CRCINA, INSERM, Université de Nantes, Université d’Angers, Angers, France; 2 LabEx ImmunoGraftOnco, Angers-Nantes, France; 3 Laboratoire d’Immunologie et Allergologie, Centre Hospitalier Universitaire, Angers, France; 4 Service de Pneumologie, Centre Hospitalier Universitaire, 4 rue Larrey, 49000 Angers, France; 5 MINT, Université d’Angers, UMR INSERM 1066 CNRS 6021, Angers, France; 6 Département de Pathologie Cellulaire et Tissulaire, Laboratoire d’Histopathologie-Cytopathologie, Centre Hospitalier Universitaire, Angers, France; 7 CRCINA, INSERM, Université d’Angers, Université de Nantes, Nantes, France

**Keywords:** Lung cancer, Sarcoid-like reaction, Nivolumab

## Abstract

**Background:**

Immune checkpoint inhibitors (ICI) target T cell inhibitory pathways that are responsible for cancer tolerance by down-modulating immune functions. ICI have revolutionized patients care with lung cancer. Nevertheless, restoring endogenous antitumor T-cell responses can induce immune related adverse events, such as sarcoidosis.

**Case presentation:**

We report here the first case of a thoracic and cutaneous sarcoid-like reaction in a patient with a relapsing unresectable non-small cell lung cancer (NSCLC) treated with nivolumab, an anti-PD-1 mAb. The expression of PD-1 and its ligands, PD-L1 and PD-L2, was assessed by flow cytometry on peripheral blood mononuclear cells (PBMC) and compared to patients who had discontinued nivolumab therapy without having developed any immune related adverse events. PD-L1 expression was transiently increased on B cells, T cells and monocytes, whereas PD-L2 expression was not modulated. PD-1 was transiently undetectable when PD-L1 was maximal, before returning to basal level. Sarcoidosis spontaneously resolved, without corticotherapy.

**Conclusion:**

This case sheds the light on a complex regulation of PD-L1 expression in vivo on PBMC after nivolumab arrest and triggers the question of monitoring the expression of immune checkpoint on immune cells during and after treatment with ICI.

## Background

Lung cancer is the first and second cause of cancer mortality in men and women, respectively, thus representing an important health problem that needs new therapeutic solutions. Immune checkpoint inhibitors (ICI), consisting in neutralizing antibodies (Ab) targeting co-inhibitory molecules to restore T cell activation, have revolutionized cancer therapy. Anti-PD-1 Abs have emerged as powerful weapons. Durable objective responses following anti-PD1 Ab therapy in patients with non-small-cell lung cancer (NSLC), accompanied by extended overall survival compared with conventional therapies, supported recent regulatory approvals by the US Food and Drug Administration for the use of nivolumab and pembrolizumab, two different anti-PD1 Abs, in these indications. Nevertheless, patients treated with ICI can develop immune related adverse events, such as dermatological toxicities, as a consequence of counteracting T cell inhibition. We report here a case of sarcoidosis in a 56-year-old patient with unresectable NSLC and treated with nivolumab.

PD-1 and PD-1 ligands expression was assessed on peripheral blood mononuclear cells (PBMC) of the patient, at different time-points (days 42, 56, 147 and 251) after nivolumab arrest.

## Case presentation

**Fig. 1:**
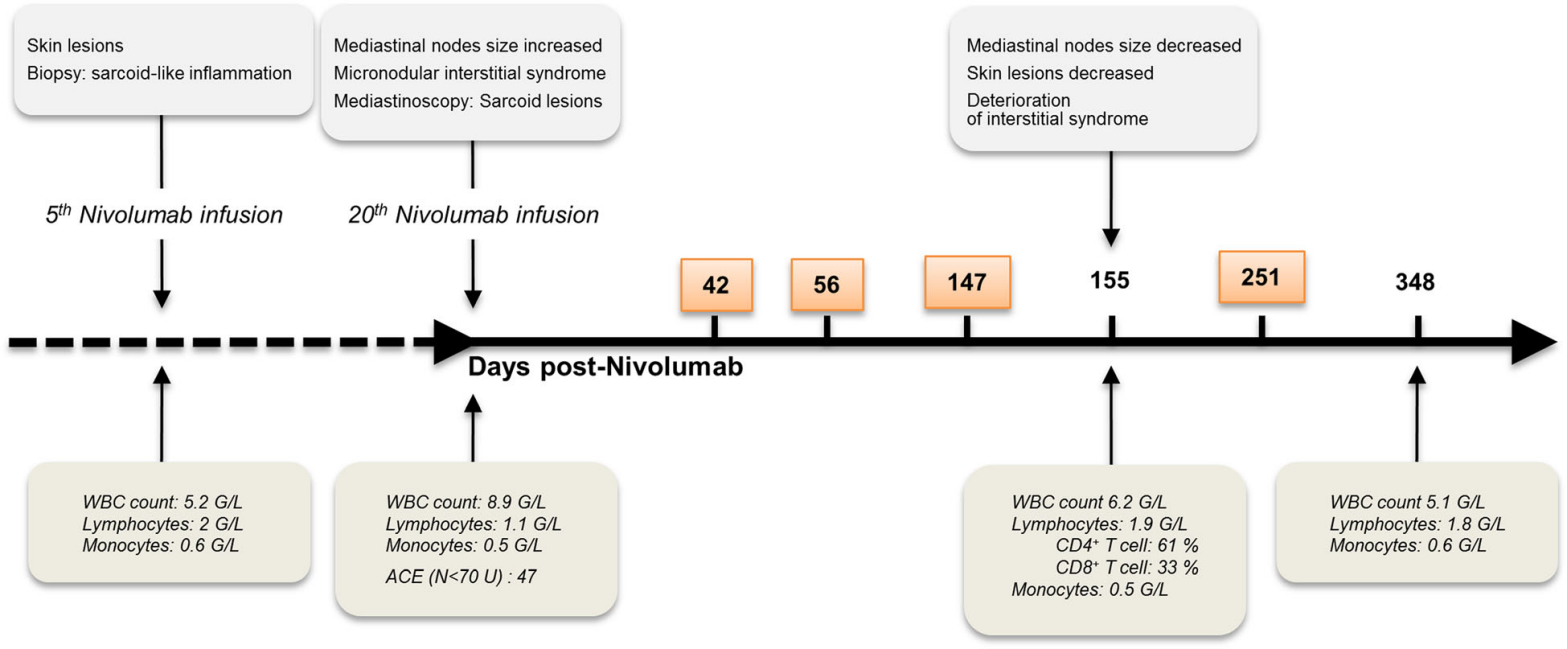
Timeline of clinical and biological analysis. Timeline reports the clinical signs of sarcoid-like reaction (upper part) and the biological parameters (lower part) of the index case. Boxed numbers, time of analysis by flow cytometry of PD-1 and PD-L1 expression by PBMC of the index case. *WBC* White blood cells; *ACE* Angiotensin converting enzyme

Nivolumab was initiated to treat a 56-year-old woman, with unresectable NSCLC who initially received an adjuvant chemotherapy consisting of 4 cycles of cisplatin-pemetrexed. New lesions appeared and were classified pT3N0M0. Nivolumab therapy was then initiated. Figure 1 summarizes the key clinical and immunological data of the index case as well as the timeline of immune checkpoint analysis.

**Fig. 2:**
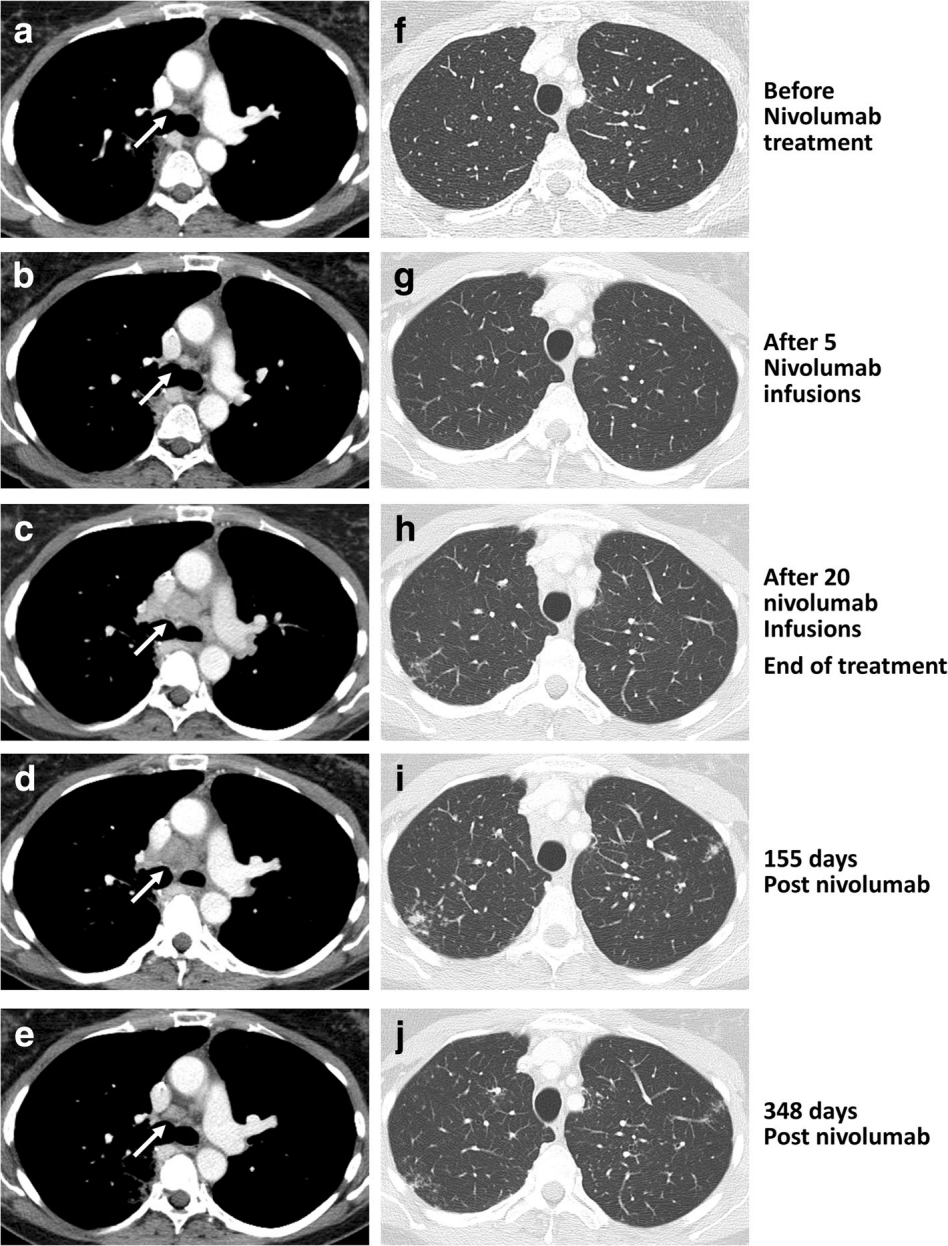
Measure of the size of mediastinal lymph nodes and interstitial syndrome. Plain chest computed tomography of the index case was carried out at different time before and after treatment discontinuation. The mediastinal lymph nodes (white arrows) measurement was achieved with a mediastinal-window setting (left panels, **a**-**e**) whereas interstitial syndrome was evaluated with a lung-window setting (right panels, **f**-**j**)

**Fig. 3:**
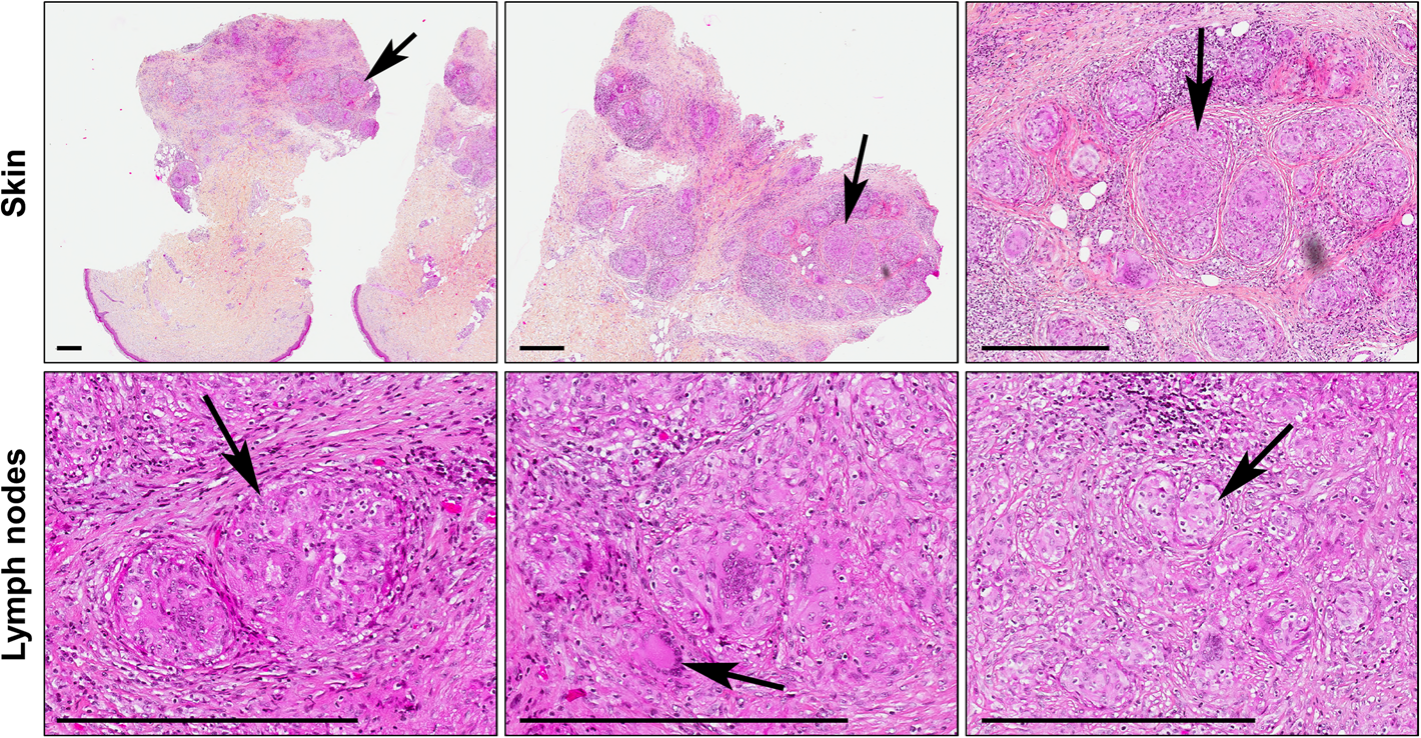
Histology of skin and lymph nodes lesions. Skin (upper panels, collected at the 5th nivolumab infusion) and thorax lymph nodes (lower panels, collected at the 20th nivolumab infusion) were analyzed by immunohistochemistry (hematoxylin phloxine saffron staining). Left to right panels, higher magnifications (scale bar, 400 μm). Black arrows correspond to lesions

No sign of SLR was detectable before the treatment with nivolumab (Fig. 2a and f). A partial response was observed after 5 infusions of nivolumab as suggested by CT scan (Fig. 2b and g). Skin lesions appeared after 5 nivolumab infusions (Fig. 3), then mediastinal nodes size started to increase and a micronodular interstitial syndrome was observed (Fig. 2c and h) after 20 nivolumab infusions. Biopsies showed epithelioid cells and Langhans multinucleated giant cells without necrosis, microorganisms or refringent bodies, compatible with sarcoid-like inflammation (Fig. 3). Tumor cells, alcohol-acid resistant bacilli and fungus or parasite were not detected (data not shown). Nivolumab was then discontinued. According to RECIST criteria, the patient had a partial response at this time.

Mediastinoscopy revealed sarcoid lesions. Mediastinal nodes sizes (Fig. 2d) and skin lesions were decreased 155 days later (data not shown), while the interstitial syndrome had deteriorated (Fig. 2i). Bronchoalveolar lavage showed hypercellularity comprising 41% of lymphocytes without pathogens or cancer cells (data not shown). 348 days later, CT scan showed normal mediastinal nodes and regression of the interstitial syndrome (Fig. 2e and j).

**Fig. 4:**
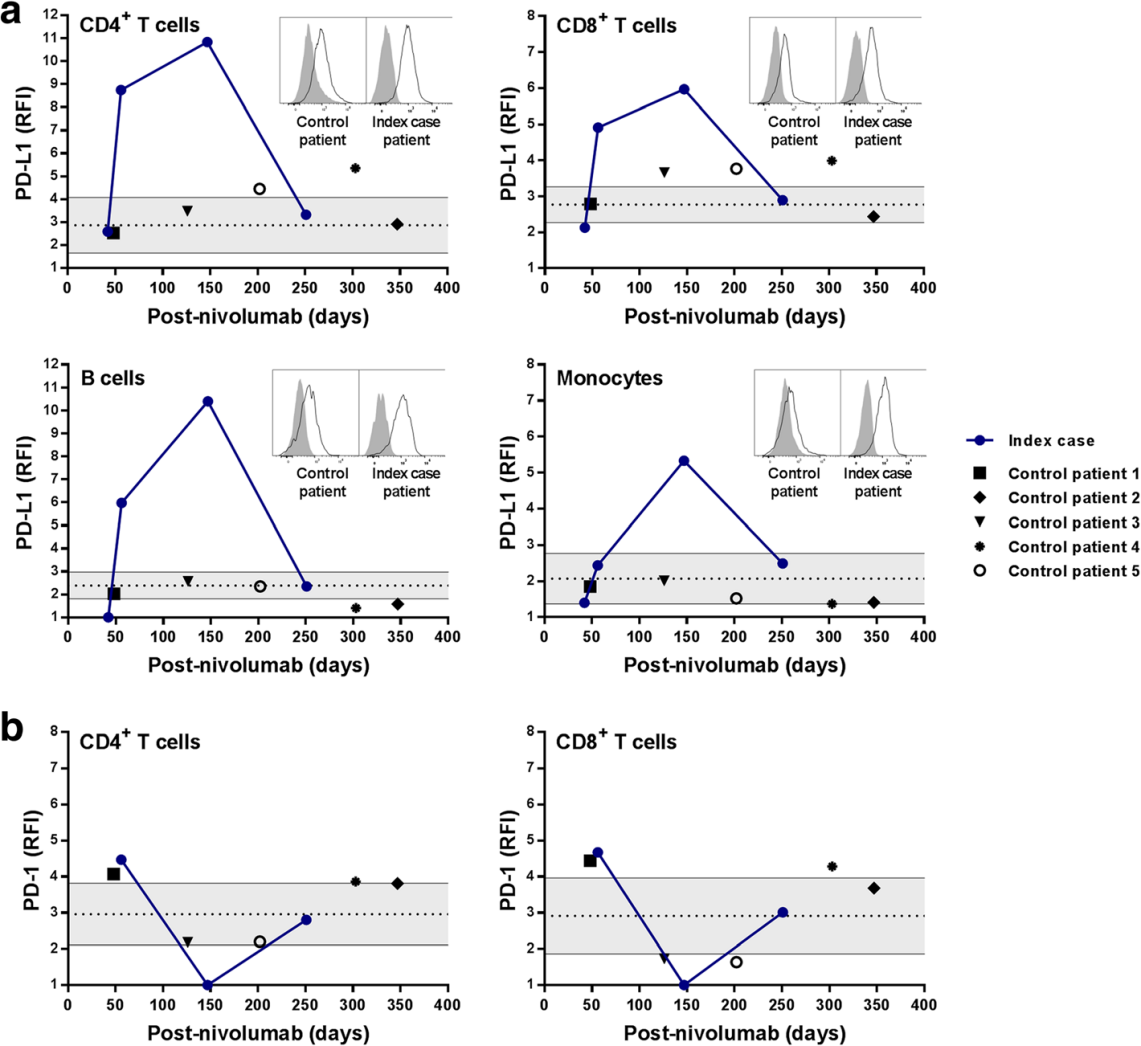
Analysis of PD-L1 and PD-1 expression on PBMC. PD-L1 (**a**) and PD-1 (**b**) expression was analyzed by flow cytometry on monocytes (CD14^+^ CD3^−^ CD19^−^), B cells (CD19^+^ CD14^−^ CD3^−^), CD4^+^ T cells (CD3^+^ CD4^+^ CD8^−^ CD19^−^ CD14^−^) and CD8^+^ T cells (CD3^+^ CD8^+^ CD4^−^ CD19^−^ CD14^−^) from the index case and from control patients at different times after the nivolumab treatment was stopped. Results are expressed as relative fluorescence intensity (RFI) defined as the ratio of specific fluorescence (median fluorescence of cells incubated with the anti-PD-L1 or anti-PD-1 Abs) over non-specific fluorescence (median fluorescence of cells incubated with the isotype control Ab). Each value from the time course analysis of the patients is plotted. The grey area represents the 95% confidence interval of the level of PD-L1 or PD-1 expression on cells from 8 healthy subjects. Inserts, flow cytometry histograms for a representative control patient (left panels) and for the index case patient (right panels, climax of the PD-L1 expression)

The expression of PD-1, PD-L1 and PD-L2 was analyzed on PBMC at various time-points after stopping nivolumab (defined as day 0). An important increase of PD-L1 expression was observed on B and T cells at d56 with a peak at d147, compared to other patients treated with nivolumab without relevant immune related reactions (Fig. 4a). An elevated expression of PD-L1 was observed on monocytes at d147 (Fig. 4a). PD-L1 expression by PBMC of the index case returned to basal levels at d251 (Fig. 4a). If we hypothesize that the increase of PD-L1 expression was consecutive to a rebound effect after stopping the treatment, we would have observed a similar increase in patients treated with nivolumab that did not exhibit immune related events. Moreover, expression of PD-1 on T cells was punctually undetectable at d147 at a time when PD-L1 expression was maximal (Fig. 4b). No marked change of PD-L2 expression was observed. Intriguingly, the increased PD-L1 expression was evidenced only from day 56 to day 147 after nivolumab arrest (Fig. 4a). This observation suggests that an elevated expression of PD-L1 consecutive to blocking PD-1/PD-L1 interaction can be associated with sarcoid-like reaction (SLR).

## Discussion and concluding remarks

Immune checkpoint inhibitors have revolutionized the therapeutic landscape in oncology. However, several immune-related adverse events have been described, including damages in skin, gastrointestinal tract, kidney and nervous system. Among them, 11 cases of sarcoidoisis-like syndroms have been reported during melanoma treatment with ipilimumab, an anti-CTLA-4 mAb, used alone or in combination with nivolumab [1, 2]. More recently, studies reported that the inhibition of the PD-1 / PD-1 L axis may also induce such adverse effects. Sarcoid development has also been reported in 2 melanoma patients treated with nivolumab [3, 4] and in one melanoma patient treated with an anti-PD-L1 mAb [5]. SLR were also reported in 3 sarcoma [6] and one Hodgkin’s disease patient [7] treated with pembrolizumab. In lung cancer, one study reported a case of sarcoidosis with ipilumimab plus nivolumab [2]. A recent publication reported nivolumab-related cutaneous sarcoidosis in a patient with lung adenocarcinoma [8]. We report here the first case of thoracic and cutaneous sarcoidosis in a patient with lung cancer and treated with nivolumab.

To overcome the side effects of ICI, immunosuppressive drugs, such as steroids, are usually used for a short period of time, with or without retracting ICI. Interestingly, in this case, SLR resolved in the absence of corticotherapy. In support, sarcoidosis spontaneously resolved in sarcoma patients treated with pembroluzimab [6].

It is interesting to note that the rapid decrease of PD-L1 expression precedes the resolution of SLR. One study has reported a local upregulation of PD-L1 expression in sarcoidosis and suggested that PD-1 blockade could constitute a therapeutic target in sarcoidosis [9]. The increase of PD-L1 may also result from the unlocking of cytokine production by activated T cells present in sarcoid lesions as well as in the peripheral blood [10]. Indeed, restoring the functions of hyper-activated TCR^high^, and especially the secretion of IFN-γ, may favor sarcoidosis-like reaction [11].

Various lines of evidence support a role of the PD-1/PD-L1 pathway in the prevention and downregulation of SLR: (a) SLR can occur during anti-PD-1/PD-L1 and -CTLA-4 Ab therapies [1, 2], (b) we and others [6] have reported a favorable outcome after nivolumab discontinuation without corticosteroid, (c) we report that PD-L1 overexpression is concomitant with regression of clinical and radiological signs of SLR and (d) PD-L1^high^ B cells have been shown to reduce inflammation [12]. Collectively, our observations suggest that a dysregulated expression of PD-1 and PD-1 L is associated with clinical signs of sarcoidosis-like syndromes.

In conclusion, this study evidences an original case of sarcoidosis-like syndrome under nivolumab with spontaneous regression after treatment arrest, in the absence of corticotherapy. It also shows that, in a patient exhibiting autoimmune related effects, nivolumab arrest is associated with a delayed and complex modulation of PD-1 and PD-L1 expression on immune cells, which may contribute to restore immune homeostasis. This observation opens the question to which extent PD-1 expression in vivo controls PD-L1 expression.
